# PHES scores have limited impact on the risk of overt HE in patients with minimal HE

**DOI:** 10.1097/HC9.0000000000000438

**Published:** 2024-05-03

**Authors:** Simon Johannes Gairing, Chiara Mangini, Lisa Zarantonello, Elise Jonasson, Sven Danneberg, Philippe Sultanik, Peter Robert Galle, Stefania Gioia, Joachim Labenz, Anna S. Lok, Jens Uwe Marquardt, Mette Munk Lauridsen, Patricia P. Bloom, Dominique Thabut, Silvia Nardelli, Sara Montagnese, Christian Labenz

**Affiliations:** 1 Department of Internal Medicine I, University Medical Center of the Johannes Gutenberg-University, Mainz, Germany; 2 Cirrhosis Center Mainz (CCM), University Medical Center of the Johannes Gutenberg-University, Mainz, Germany; 3 Department of Medicine, University of Padova, Padova, Italy; 4 Department of Gastroenterology and Hepatology, Hospital of South West Jutland, Esbjerg, Denmark; 5 Department of Medicine I, University Hospital Schleswig-Holstein, Lübeck, Germany; 6 Service d’hépato-gastroentérologie, Sorbonne Université, Hôpital Pitié-Salpêtrière Assistance Publique Hôpitaux de Paris, Paris, France; 7 Department of Translational and Precision Medicine, Sapienza University of Rome, Rome, Italy; 8 Department of Medicine, Diakonie Hospital Jung-Stilling, Siegen, Germany; 9 Department of Medicine, Division of Gastroenterology and Hepatology, University of Michigan, Ann Arbor, Michigan, USA; 10 Chronobiology Section, Faculty of Health and Medical Sciences, University of Surrey, Guildford, UK

## Abstract

**Background:**

Minimal hepatic encephalopathy, defined by the portosystemic hepatic encephalopathy score (PHES), is associated with a higher risk of subsequent OHE. It remains unclear if there is a stepwise increase in OHE risk with worse PHES results.

**Methods:**

In this multicenter study, patients with minimal hepatic encephalopathy, as defined by abnormal PHES, were followed for OHE development.

**Results:**

In all, 207 patients were included. There was no stepwise increase in OHE risk with worse PHES results.

**Conclusions:**

Abnormal PHES is associated with a higher OHE risk, but we found no stepwise increase in OHE risk with worse PHES results below the established cutoff.

## INTRODUCTION

HE is a severe complication of cirrhosis and is associated with poor prognosis.^1^ Therefore, prevention of overt hepatic encephalopathy (OHE), and especially severe OHE requiring hospitalization, is of pivotal importance to improve quality of life and prognosis. It is well-known that patients with minimal hepatic encephalopathy (MHE) defined by the psychometric hepatic encephalopathy score (PHES-MHE) are at higher risk for developing OHE compared to patients without MHE.^2^ However, not every patient with MHE develops an episode of OHE in the near future, and accordingly, primary prophylaxis is recommended only on a case-by-case basis.^3^ Therefore, identifying high-risk patients in the subgroup of patients with MHE could be beneficial to establishing strategies to prevent OHE in routine clinical practice and for purposes of future interventional trials.^4^ This study aimed to investigate whether there is a stepwise increase in OHE risk with worse PHES results in patients with PHES-MHE.

## METHODS

This retrospective study analyzed data of 1462 patients, who underwent testing with the PHES and had available follow-up data. Patients were studied in 8 centers with expertise in diagnosing MHE: Mainz, Siegen, Lübeck (all Germany), Paris (France), Padua and Rome (Italy), Esbjerg (Denmark), and Ann Arbor (Michigan, USA). For this study, we only included patients with PHES-MHE, no history of OHE, and no prescription for lactulose or rifaximin (excluded: 959 without PHES-MHE, 212 with PHES-MHE but with a history of OHE, 84 with a prescription for lactulose or rifaximin). Additional details of this study regarding the diagnosis of cirrhosis and all exclusion criteria for each center can be found in previous publications based on the same cohort (more details in the Supplemental Methods, http://links.lww.com/HC9/A879).^2,5^

All patients were examined at the respective hospitals to rule out signs of OHE, and MHE was subsequently diagnosed by PHES (more details are provided in the Supplemental Methods, http://links.lww.com/HC9/A879). PHES was scored at each center using the validated country-specific norms (Germany and Denmark,^6^ United States,^7^ Italy,^8^ France^9^). A score <− 4 was considered diagnostic of PHES-MHE for centers from Germany, France, and Denmark, while the centers from Italy and the United States used a score ≤− 4. For subsequent analyses and to make results between centers more comparable, we calculated a scoring system adjusted PHES scores (adjusted PHES) by dividing the PHES score by the number of subtests. As an example for German patients: if a patient had a PHES score of -12, this score was divided by the number of subscores of PHES (in Germany 6 subscores), resulting in an adjusted PHES of -2. All patients were followed up for the occurrence of OHE and liver transplantation-free survival.

The study was conducted in accordance with the ethical guidelines of the Declaration of Helsinki. For this study, we used anonymized electronic medical records without directly identifiable data. According to German regulations and the recommendations of the Ethics Committee of the Landesärztekammer Rheinland-Pfalz, no ethical approval is required. Regarding a subset of patient data that were recorded in a prospective setting, the respective study protocols were approved by the Ethics Committees of the respective centers, and informed consent was obtained from each participant.

Statistical analyses and graphic design were performed with R 4.1.3 (R Core Team, 2022). We examined the correlation between OHE risk and the adjusted PHES as a continuous variable and as a categorical variable divided into quartiles. Death/liver transplantation was classified as a competing event in the multi-state model analyses.

Detailed information on the statistical analyses is provided in the Supplemental Methods, http://links.lww.com/HC9/A879.

## RESULTS

Baseline characteristics of the patient cohort (n = 207) are displayed in Supplemental Table S1, http://links.lww.com/HC9/A879. The median adjusted PHES was −1.20 (range −2.67, −0.80). The median follow-up time was 18.5 months (95% CI: 13.9–20.6). In total, 59 (29%) patients developed an OHE episode during follow-up. Additionally, 41 (20%) patients reached the end point of death/liver transplantation during follow-up before an episode of OHE.

**FIGURE 1:**
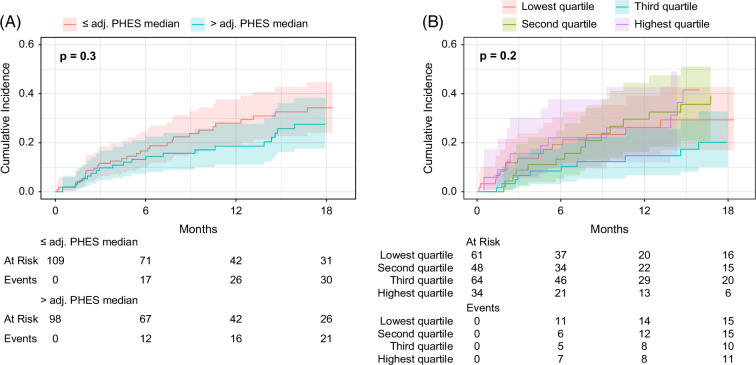
Cumulative overt HE (OHE) incidences. Cumulative OHE incidences in the total cohort stratified by (A) adjusted PHES median and (B) adjusted PHES quartiles. Abbreviations: Inf, infinity; OHE, overt hepatic encephalopathy; PHES, Psychometric Hepatic Encephalopathy Score.

To analyze the association between worse results in the adjusted PHES and the risk of OHE development, the cohort was split for 2 separate analyses: (i) into 2 groups according to the median and (ii) into 4 groups according to the quartiles of the results in adjusted PHES. In both analyses, the cumulative OHE incidences did not differ significantly between patients with poorer or better results in PHES (Figure 1A-B). In multivariable competing risk regression analyses, adjusted PHES as a continuous variable (sHR 1.05, 95% CI: 0.55–2.01, *p* = 0.9) or categorized into quartiles (each *p* ≥ 0.2) were not associated with the development of OHE (Supplemental Table S2, http://links.lww.com/HC9/A879). No association was observed in multivariable models only including patients with Child-Pugh A or B/C (Supplemental Table S2, http://links.lww.com/HC9/A879) either.

## DISCUSSION

MHE, defined by an abnormal PHES, is associated with a higher risk of developing OHE.^2^ Whether PHES results below these cutoffs are associated with a further increase in the risk of future OHE episodes, as well as the rationale for this study, was unknown. In this multicenter study, we found no stepwise increase in OHE risk with worse PHES results below the cutoff defining MHE.

Identification of high-risk patients for OHE development is important for the management of patients with cirrhosis^4^ and MHE is a well-known risk factor for OHE; however, our study demonstrates that within the group of patients with MHE, worse results in PHES cannot be taken to reflect a higher OHE risk. This may relate to the fact that cognitive function varies from patient to patient, and PHES results are not only affected by MHE. This means that one may not know a patient’s “baseline” cognitive performance in PHES by testing the patient only once. Therefore, an important research question might be whether serial testing, particularly in patients with results below the cutoff defining MHE, and detection of subsequent changes in PHES results might be more informative for predicting OHE risk.^10^ Future studies should focus on serial testing and determine potential thresholds for a predictive delta of changes in PHES. In the context of 1-time testing with PHES, our study highlights that additional instruments are needed for more granular risk stratification. Here, biomarkers could be a promising adjunct, as recently demonstrated for ammonia.^11^ The combination of a biomarker and a phenotypic index may also be a reasonable and promising route. Importantly, our findings should not deter physicians from considering the effects of varying degrees of PHES abnormalities on everyday functioning and quality of life, even within the MHE category, and especially from treating patients with MHE.

A limitation of this study that has to be acknowledged is that we cannot account for the potential initiation of HE treatment during follow-up, as this piece of information was not available in this data set. Additionally, this database lacks information on, for example, non-liver–related comorbidities and we are therefore unable to adjust for these factors. Another limitation is that potentially not all OHE episodes might be captured if they were not registered in the respecting hospitals participating in this study.

In conclusion, our study provides evidence that in patients with PHES-MHE, there is no stepwise increase in OHE risk with worse PHES results.

## Supplementary Material
