# Supplementary material for: PHES scores have limited impact on the risk of overt HE in patients with minimal HE
Source: Hepatol Commun. 2024 May 3;8(5):e0438. doi: 10.1097/HC9.0000000000000438 (PMC11073767; doi:10.1097/HC9.0000000000000438)
Supplement: Supplementary file 1 [file hc9-8-e0438-s001.docx]

**PHES scores have limited impact on the risk of overt hepatic encephalopathy in patients with minimal hepatic encephalopathy**

*Running head: PHES and hepatic encephalopathy*

Simon Johannes Gairing, MD^1,2^, Chiara Mangini, MD^3^, Lisa Zarantonello, PhD^3^, Elise Jonasson Nielsen, MD^4^, Sven Danneberg, MD^5^, Philippe Sultanik, PhD^6^, Peter Robert Galle, MD^1,2^, Stefania Gioia, MD^7^, Joachim Labenz, MD^8^, Anna S. Lok, MD^9^, Jens Uwe Marquardt, MD^5^, Mette Munk Lauridsen, MD^4^, Patricia P. Bloom, MD^9^, Dominique Thabut, MD^6^, Silvia Nardelli, MD^7^, Sara Montagnese, MD, PhD^3,10^, Christian Labenz, MD^1,2^

**Table of contents**

[Supplementary methods 2](#_Toc157350215)

[Supplementary tables 4](#_Toc157350216)

# Supplementary methods

# At each center, the primary etiology of the underlying liver disease was determined according to clinical, serological, and histological findings. Diagnosis of cirrhosis was established by histology, conclusive appearance on ultrasound, elastography or radiological imaging, endoscopic features of portal hypertension, or medical history. Patients were not included in this study if they had a history of any other disease leading to mental alterations (e.g. dementia or a history of stroke) or were using illicit drugs. At some centers, patients with self-reported ongoing (mild) alcohol consumption were included provided that they were not under the effects of alcohol during testing with PHES.

# Assessment of MHE

All patients were examined at the respective hospitals to rule out signs of OHE and MHE was subsequently diagnosed by PHES. PHES is a paper-and-pencil testing battery including five subtests:

- number connection test A
- number connection test B
- serial dotting test
- digit symbol test
- line tracing test

# Cut-offs defining MHE vary between countries. According to established norms, a score < -4 was considered diagnostic of PHES-MHE for centers from Germany, France and Denmark, while the centers from Italy and the US used a score ≤ -4.

**Follow-up evaluation**

Patients were followed during regular clinic visits or via electronic chart review at the respective centers for the occurrence of OHE. The presence of OHE was diagnosed after detailed neurological examination according to the West-Haven-Criteria by an experienced hepatologist. Moreover, patients were followed for the occurrence of either death or liver transplantation. Patients who did not reach the endpoint of death/liver transplantation were censored at the date of last contact.

# Supplementary statistics

Baseline characteristics were calculated with the {table1} R package (v1.4.3; Benjamin Rich 2023). Continuous variables are given as median with range, categorial variables as frequencies with percentages. Groups were compared by an unpaired t-test, a Mann-Whitney U test or a chi-squared test, as appropriate. The {prodlim} R package (v2019.11.13, Thomas A. Gerds 2019) was used to calculate the median follow-up time (reverse Kaplan-Meier method). The {tidycmprsk} R package (v0.2.0, Daniel D. Sjoberg and Teng Fei 2022) were used to fit both cumulative incidence functions (CIF) and Fine and Gray competing risk regression analyses. Gray’s test was calculated to test for differences between groups in CIFs. To analyze the risk of OHE development, death and liver transplantation were defined as competing events. Only complete cases were included in the analyses. P‑values < 0.05 were considered significant.

# Supplementary tables

**Table S1. Demographics and clinical characteristics of the study cohort.**

|  | **Below adjusted PHES median**  **[-Inf, -1.2] (n = 109)** | **Above adjusted PHES median**  **(-1.2, Inf] (n = 98)** | **P-value** |
| --- | --- | --- | --- |
| **Age (years)** |  |  |  |
| Median [Min, Max] | 66.0 [35.0, 86.0] | 61.0 [27.0, 80.0] | 0.03 |
| **Gender** |  |  |  |
| male | 76 (69.7%) | 66 (67.3%) | 0.8 |
| female | 33 (30.3%) | 32 (32.7%) |  |
| **Etiology** |  |  |  |
| Alcohol | 47 (43.1%) | 44 (45.4%) | 0.2 |
| Viral | 41 (37.6%) | 26 (26.8%) |  |
| Others/mixed | 21 (19.3%) | 27 (27.8%) |  |
| Missing | 0 (0%) | 1 (1.0%) |  |
| **MELD score** |  |  |  |
| Median [Min, Max] | 11.5 [6.00, 34.0] | 11.0 [6.00, 31.0] | 0.3 |
| Missing | 1 (0.9%) | 1 (1.0%) |  |
| **ALBI score** |  |  |  |
| Median [Min, Max] | -1.99 [-3.28, 0.143] | -2.05 [-3.40, -0.154] | 0.4 |
| Missing | 24 (22.0%) | 19 (19.4%) |  |
| **Child Pugh** |  |  |  |
| A | 47 (43.1%) | 39 (40.6%) | 0.5 |
| B | 41 (37.6%) | 43 (44.8%) |  |
| C | 21 (19.3%) | 14 (14.6%) |  |
| Missing | 0 (0%) | 2 (2.0%) |  |
| **ALBI grade** |  |  |  |
| 1 | 18 (21.2%) | 16 (20.3%) | 0.4 |
| 2 | 44 (51.8%) | 48 (60.8%) |  |
| 3 | 23 (27.1%) | 15 (19.0%) |  |
| Missing | 24 (22.0%) | 19 (19.4%) |  |
| **Adjusted PHES** |  |  |  |
| Median [Min, Max] | -1.67 [-2.67, -1.20] | -1.00 [-1.17, -0.800] | < 0.001 |
| **History of ascites** |  |  |  |
| no | 53 (48.6%) | 43 (44.8%) | 0.7 |
| yes | 56 (51.4%) | 53 (55.2%) |  |
| Missing | 0 (0%) | 2 (2.0%) |  |
| **Sodium (mmol/L)** |  |  |  |
| Median [Min, Max] | 138 [112, 144] | 137 [124, 147] | 0.9 |
| Missing | 13 (11.9%) | 9 (9.2%) |  |
| **Creatinine (mg/dL)** |  |  |  |
| Median [Min, Max] | 0.912 [0.400, 6.73] | 0.830 [0.385, 1.86] | 0.02 |
| Missing | 1 (0.9%) | 1 (1.0%) |  |
| **Bilirubin (mg/dL)** |  |  |  |
| Median [Min, Max] | 1.28 [0.100, 29.4] | 1.26 [0.270, 46.5] | 0.5 |
| Missing | 2 (1.8%) | 1 (1.0%) |  |
| **Albumin (g/L)** |  |  |  |
| Median [Min, Max] | 33.0 [18.0, 47.0] | 34.1 [17.0, 51.1] | 0.2 |
| Missing | 12 (11.0%) | 10 (10.2%) |  |
| **INR** |  |  |  |
| Median [Min, Max] | 1.25 [0.900, 3.23] | 1.27 [0.900, 2.70] | 0.7 |
| Missing | 1 (0.9%) | 2 (2.0%) |  |
| **Platelets (per nL)** |  |  |  |
| Median [Min, Max] | 113 [36.0, 322] | 111 [41.0, 551] | 0.7 |
| Missing | 20 (18.3%) | 17 (17.3%) |  |

Percentages refer to the patients with available data. Abbreviations: MELD, model for end-stage liver disease; ALBI, albumin-bilirubin; PHES, psychometric hepatic encephalopathy score; INR, international normalized ratio.

**Table S2. Competing risk regression analysis for the development of OHE.**

| **Variable** | **n** | **sHR** | **95% CI** | **p-value** |
| --- | --- | --- | --- | --- |
| **Adjusted PHES as continuous variable**  **(total cohort) *** |  |  |  |  |
| Adjusted PHES (cont.) | 184 | 1.05 | 0.55, 2.01 | 0.9 |
| Albumin (g/L) | 184 | 0.95 | 0.91, 1.00 | 0.036 |
| MELD | 184 | 1.05 | 0.99, 1.11 | 0.11 |
| Age (years) | 184 | 1.02 | 1.00, 1.04 | 0.12 |
| **Adjusted PHES split into quartiles**  **(total cohort) *** | | | | |
| Adjusted PHES (quart.) |  |  |  |  |
| [-Inf, -1.67] | 53 | — | — |  |
| (-1.67, -1.2] | 43 | 1.00 | 0.49, 2.07 | >0.9 |
| (-1.2, -1] | 61 | 0.61 | 0.27, 1.34 | 0.2 |
| (-1, Inf] | 27 | 1.48 | 0.67, 3.27 | 0.3 |
| Albumin (g/L) | 184 | 0.95 | 0.91, 1.00 | 0.044 |
| MELD | 184 | 1.04 | 0.98, 1.10 | 0.2 |
| Age (years) | 184 | 1.02 | 0.99, 1.04 | 0.2 |
| **Only patients with Child-Pugh A** |  |  |  |  |
| Adjusted PHES (cont.) | 71 | 0.66 | 0.18, 2.38 | 0.5 |
| Albumin (g/L) | 71 | 1.00 | 0.89, 1.12 | >0.9 |
| MELD | 71 | 0.90 | 0.70, 1.16 | 0.4 |
| Age (years) | 71 | 1.02 | 0.95, 1.09 | 0.6 |
| **Only patients with Child-Pugh B/C** |  |  |  |  |
| Adjusted PHES (cont.) | 113 | 0.98 | 0.44, 2.19 | >0.9 |
| Albumin (g/L) | 113 | 0.97 | 0.91, 1.02 | 0.2 |
| MELD | 113 | 1.04 | 0.98, 1.10 | 0.2 |
| Age (years) | 113 | 1.02 | 1.00, 1.05 | 0.11 |

Abbr.: sHR, subdistribution hazard ratio; CI, confidence interval;

PHES, psychometric hepatic encephalopathy score; MELD, Model for End-stage Liver Disease;

cont., continuous.

*23 patients were excluded from the competing risk regression analysis due to missing data.
